# Genes related to redox and cell curvature facilitate interactions between *Caulobacter* strains and *Arabidopsis*

**DOI:** 10.1371/journal.pone.0249227

**Published:** 2021-04-01

**Authors:** Louis Berrios, Bert Ely

**Affiliations:** Department of Biological Sciences, University of South Carolina, Columbia, SC, United State of America; Universidade de Coimbra, PORTUGAL

## Abstract

Bacteria play an integral role in shaping plant growth and development. However, the genetic factors that facilitate plant-bacteria interactions remain largely unknown. Here, we demonstrated the importance of two bacterial genetic factors that facilitate the interactions between plant-growth-promoting (PGP) bacteria in the genus *Caulobacter* and the host plant *Arabidopsis*. Using homologous recombination, we disrupted the cytochrome ubiquinol oxidase (*cyo*) operon in both *C*. *vibrioides* CB13 and *C*. *segnis* TK0059 by knocking out the expression of *cyoB* (critical subunit of the *cyo* operon) and showed that the mutant strains were unable to enhance the growth of *Arabidopsis*. In addition, disruption of the *cyo* operon, metabolomic reconstructions, and pH measurements suggested that both elevated *cyoB* expression and acid production by strain CB13 contribute to the previously observed inhibition of *Arabidopsis* seed germination. We also showed that the crescent shape of the PGP bacterial strain *C*. *crescentus* CB15 contributes to its ability to enhance plant growth. Thus, we have identified specific genetic factors that explain how select *Caulobacter* strains interact with *Arabidopsis* plants.

## Introduction

Terrestrial plants and microbes have been coevolving for over 100 million years [1], and their interactions contribute to global biogeochemical cycles and agricultural fecundity [2]. Recent advances in microbial ecology have facilitated taxonomical and functional classifications of plant-associated microbes (PAMs), and core plant microbiomes (conserved microbial taxa) have begun to be identified across various plant species and diverse geographic regions [3,4]. For instance, sequence-based approaches have highlighted the abundance of *Alphaproteobacteria* species in (endosphere) and around (rhizosphere) the roots of many plant genera such as *Arabidopsis*, *Glycine*, *Hordeum*, *Panicum*, *Sorghum*, *Triticum*, and *Zea mays* across diverse geographical regions [5–12]. Pioneering work borne out of the last decade has expedited our understanding of PAMs and has highlighted the prevalence of plant-growth-promoting bacteria (PGPB) [5,8,13,14]. The seminal works of Bulgarelli et al. (2015) [5] and Lundberg et al. (2012) [8] established that the core microbiome of *Arabidopsis* assembles based primarily on the ability of its microbial members to metabolize root exudates (primarily carbon), and ‘hub strains’ tend to play integral roles in the assembly and maintenance of plant microbiomes. However, detailed functional roles for hub strains have yet to be established, and the degree to which they function as PGPB remains elusive.

Recent communications have commented on the prevalence of reductive and oxidative (redox) enzyme coding genes in the genomes of PAMs [15–18], and functional interactions between PAMs and their hosts have been further understood by implementing inoculum-based synthetic communities to explore and verify the requirement of select microbial genes for a given function (e.g., root colonization) [18,19]. Nonetheless, functional genetics approaches that seek to resolve the function of redox related activities in the context of PGPB assays have not been communicated and many reports consider only correlative data involving common PGP factors (1-aminocyclopropane deaminase (ACC deaminase), cytokinin biosynthesis, indole-3-acetic acid (IAA) production, nitrogen fixation, and phosphate solubilization) as proxies to assess the potential of a bacterial strain to enhance plant growth [20,21]. However, common PGP factors can also negatively correlate with plant fitness [22].

The genus *Caulobacter*, a member of the class *Alphaproteobacteria*, possesses many strains that have been isolated from the endosphere and rhizosphere of *Arabidopsis*, *Citrullus*, *Lavandula* and *Zea mays* [23–26], which in part implicates members of the *Caulobacter* genus as representative microbial hub species [27]. Moreover, select *Caulobacter* strains have been shown to increase plant biomass and alter root architecture relative to uninoculated conditions [22–24]. Functional roles that explain *Caulobacter*-mediated plant growth enhancement, however, have not been reported [22–24]. A recent report from Luo et al. (2019) [23] demonstrated that *Caulobacter* sp. RHG1 cells localize to regions of lateral root formation in *Arabidopsis* and increase root length and lateral root formation compared to the roots of uninoculated plants. Similarly, we previously identified six *Caulobacter* strains that could increase plant weight and root length relative to control conditions [22], and our results suggested that common PGP factors did not explain the plant growth enhancement that we observed in our system.

To identify presumptive genes that facilitate *Caulobacter*-mediated plant growth enhancement, we previously employed a genome-wide association study (GWAS) and observed that the genomes of PGP *Caulobacter* strains harbored ~2-fold more genes with predicted reactive oxygen species (ROS) scavenging functions compared to the genomes of non-PGP *Caulobacter* strains. Specifically, we observed an extra operon (*cyo*) that is predicted to code for the biosynthesis of gomphrenin-I, which is a betalain-type ROS scavenging molecule that has been shown previously to exhibit high ROS scavenging activity [28]. Since ROS act as intracellular signaling molecules and facilitate plant growth and development [29–32], we hypothesized that this additional ROS scavenging-related operon may play a role in *Caulobacter*-mediated plant growth enhancement.

Bacterial cell shape has previously been shown to facilitate adsorption and may be a prerequisite for select cellular functions (e.g., ROS scavenging for plant host). For example, Persat et al. (2014) [40] demonstrated that the curvature of *Caulobacter* cells enhances colonization in flow, albeit curvature diversity may be selected for based on the environmental context. Similarly, the spiral shape of the bacterium *Helicobacter pylori* remains a prerequisite for effective stomach colonization and subsequent pathogenesis [33]. Recent larger scale analyses have even demonstrated that spatiotemporal distributions (i.e., proximity to plant roots as a function of time) of bacterial species may be predicated on cell shape and structure [34]. However, cell curvature has yet to be examined in the context of PGP factors. Thus, we investigated *Caulobacter* cell shape in the context of plant-microbe interactions and hypothesized that the crescent shape of *C*. *crescentus* cells may contribute to the *Caulobacter*-mediated plant growth enhancement that we previously observed [22].

To test our hypotheses, we disrupted the cytochrome ubiquinol oxidase subunit 1 (EC 1.10.3-) (hereafter *cyoB*) gene in two different PGP *Caulobacter* species (*C*. *vibrioides* CB13 and *C*. *segnis* TK0059) and compared the impact that each mutant strain had on the growth of *Arabidopsis* relative to that provided by their parental strains (wild-type). To determine if cell curvature facilitates PGP factors, we compared the effect of a *creS* mutant (required for *Caulobacter* crescent cell shape) on plant growth relative to its PGP parental strain (*C*. *crescentus* CB15). In addition, ROS play critical roles during seed germination [32], and we observed previously that CB13 severely inhibits seed germination rates, but it still increases plant weight relative to that of uninoculated plants [22]. Therefore, we explored whether differential gene expression patterns of *cyoB* across PGP *Caulobacter* strains occurred. As such, we reasoned that since elevated ROS levels are required for the seed-to-seedling transition in *Arabidopsis* [35–37], and previous reports have linked increased ROS scavenging activity to seed germination suppression [38,39], CB13 may exhibit elevated *cyoB* gene expression levels relative to other PGP strains, which would suggest that CB13 may dampen ROS levels in *Arabidopsis* seeds below the required oxidative window that drives seed germination [29]. Moreover, we determined that CB13 likely inhibits *Arabidopsis* seed germination (in part) by lowering proximal pH concentrations. Taken together, our results suggest functional roles for betalain-related gene products and cell curvature regarding *Caulobacter*-mediated plant growth enhancement and demonstrate that pH reducing metabolic factors may cause CB13 to inhibit seed germination.

## Results

### *cyoB* and *creS* contribute to *Caulobacter*-mediated plant growth enhancement

**Fig 1:**
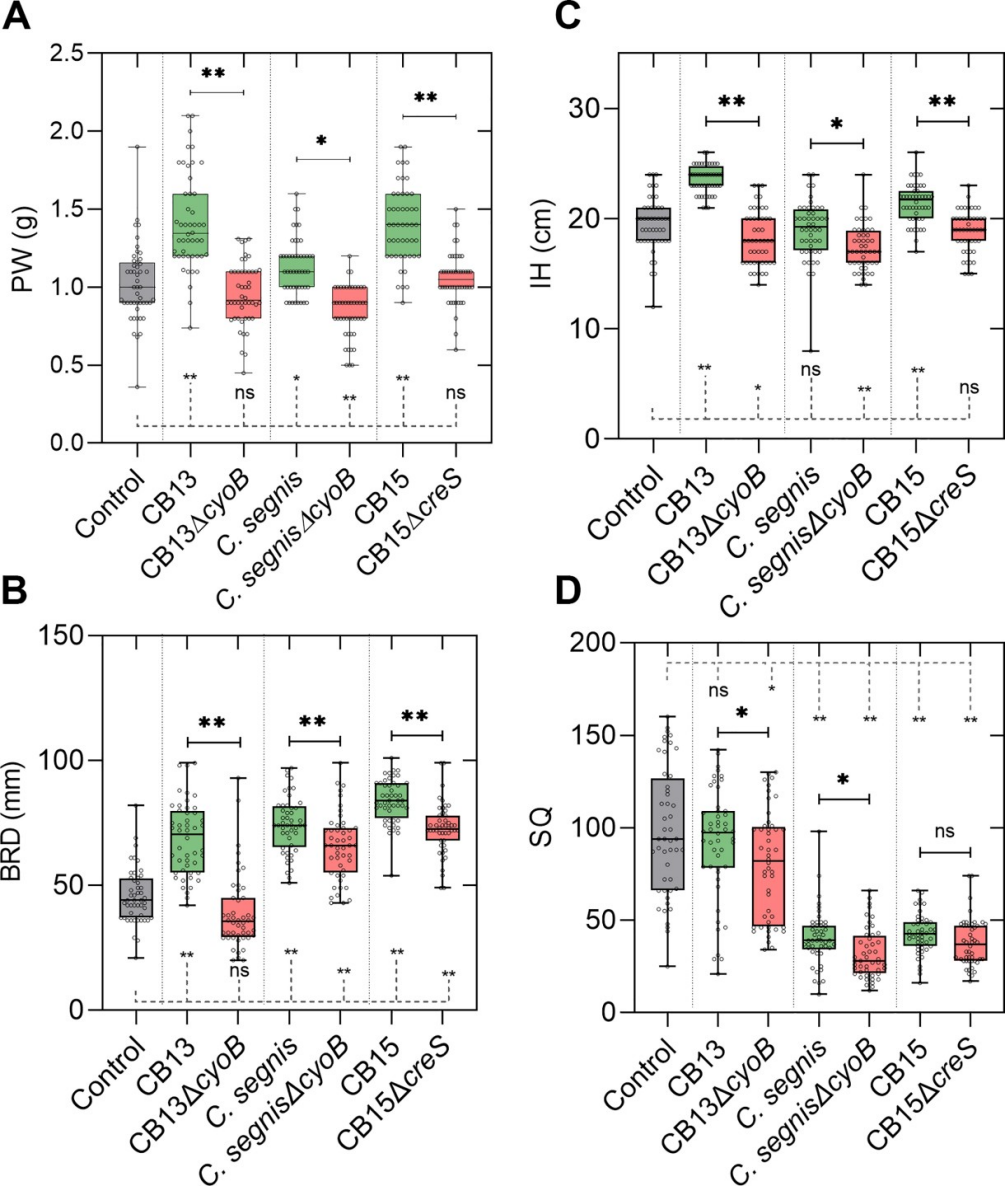
*cyoB* and *creS* contribute to *Caulobacter*-mediated plant growth enhancement. **A)** Box-and-whisker plot illustrating plant weight (PW) in grams (g), **B)** basal rosette diameter (BRD) in millimeters (mm), **C)** inflorescence height (IH) in centimeters (cm), and **D)** silique quantity (SQ) for each experimental condition. Seedlings and ungerminated seeds were transplanted from MS (Murashige and Skoog) plates to soil after 11 days. A total of 48 data points for each condition are displayed. Whiskers indicate maximum and minimum data points, and boxes span 25–75% quartiles with central bars representing the median values of the populations. A one-way ANOVA and pairwise Welch’s t-tests were performed to derive p-values. * = p < 0.01; ** = p < 0.001; ns = p > 0.01. Pairwise significance values between control and experimental conditions are connected by dashed, gray lines, and p-value matrix tables can be found in S1B Table.

Since our previous analyses suggested that the expression of betalain synthesis related genes may contribute to the *Caulobacter*-mediated plant growth enhancement that we observed in our system [22], we knocked-out the expression of the *cyoB* gene (part of the *cyoA*-*D* operon; EC 1.10.3-) that is predicted to code for an enzyme that is involved in the biosynthesis of betalain. Using homologous recombination, we disrupted the function of the *cyoB* gene in two *Caulobacter* strains, *C*. *vibrioides* CB13 (CB13Δ*cyoB*) and *C*. *segnis* TK0059 (*C*. *segnis*Δ*cyoB*) to subsequently test our hypothesis that a functional *cyo* operon is a PGP factor for more than one *Caulobacter* species. Operationally defining plant growth enhancement as increased plant weight (PW), we observed that both CB13Δ*cyoB* and *C*. *segnis*Δ*cyoB* were unable to significantly enhance plant growth relative to control conditions and their parental strains (Fig 1A and S1A and S1B Table).

**Fig 2:**
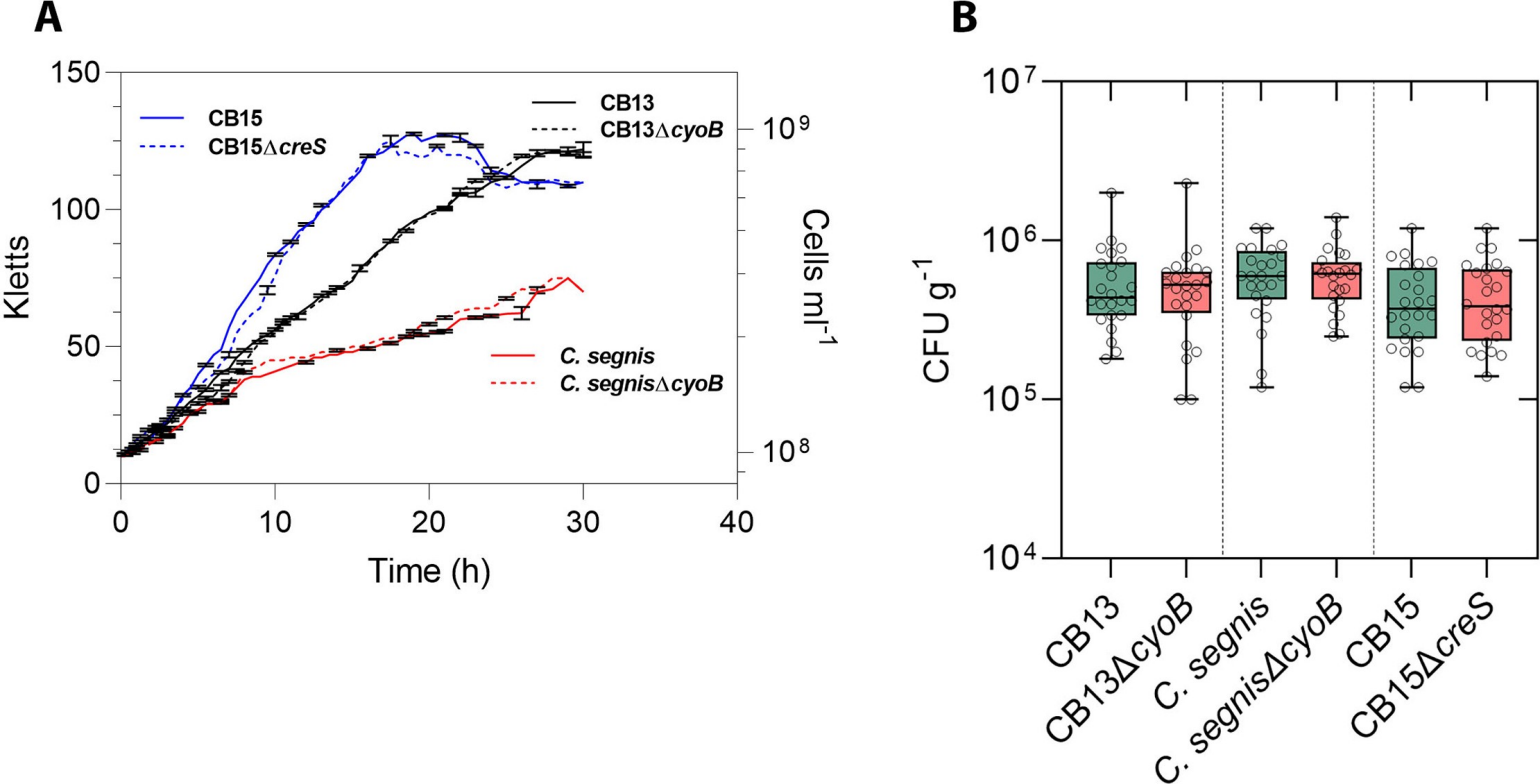
*Caulobacter* strain growth curve and re-isolation data. **A)** Replicate values (n = 3) are displayed for each timepoint. **B)** Colony-forming units (CFUs) per gram of soil are displayed for each condition. Bacteria were recovered from 12 soil samples after plant growth across each condition for both independent experiments (n = 2). The box and whisker plots include all data points. Whiskers indicate maximum and minimum data points, and boxes span 25–75% quartiles with central bars representing the median values of the populations. The raw data are in S2 Table (ns = p > 0.01).

To tease out differential effects on specific *Arabidopsis* anatomical features as a result of bacterial cell inoculation, we measured the basal rosette diameter (BRD), inflorescence height (IH), and silique quantity (SQ) and then analyzed these parameters among inoculum conditions. Consistent with our PW data, the mutant strains had little impact on BRD, IH, and SQ relative to the control plants (Fig 1B–1D and S1B Table). The one exception was that seeds that were inoculated with CB13Δ*cyoB* cells were unable to increase BRD relative to control conditions, but seeds that were inoculated with *C*. *segnisΔcyoB* cells were still able to enhance BRD relative to control conditions (Fig 1B). Although changes in SQ were observed between parental and mutant strains, none of the strains increased SQ relative to the control conditions (Fig 1D), which aligns with our previous analyses [22]. Prior to using the mutant constructs for plant bioassays (Fig 1), we ensured that neither mutant incurred obvious growth defects relative to their parental strains by measuring the growth rates of each assayed *Caulobacter* strain under low aeration conditions (Fig 2A) and moderate aeration conditions (growth on PYE agar plates at ambient O_2_ concentrations). Since differences in growth rates (cell density in PYE broth and colony forming rates on PYE plates) were not observed between mutant strains and their corresponding parental strains, and our bacterial cell re-isolation assays suggested that the observed differences in growth stimulation were likely not related to differential bacterial cell growth dynamics in the soil (Fig 2B and S2 Table), our data demonstrate the importance of a functional *cyoB* gene in the context of *Caulobacter*-mediated plant growth enhancement in two different *Caulobacter* species.

Since bacterial cell shape has been linked to colonization abilities [33,40], and *Caulobacter* cells can colonize plant roots both in artificial environments [23] and in natural environments [41], we tested whether the cell curvature of *Caulobacter* cells (i.e., using CB15 as a proxy for PGP *Caulobacter* strains since cell curvature is a conserved feature among the *Caulobacter* strains that we previously tested [22]) contributes to *Caulobacter*-mediated plant growth enhancement by conducting our plant bioassays with CB15Δ*creS* (rod shaped as opposed to the typical crescent shape of *C*. *crescentus* cells). We observed that plants grown to maturation in the presence of CB15Δ*creS* cells were significantly smaller (PW, BRD) than those grown in the presence of CB15 cells (Fig 1A–1C and S1A and S1B Table). However, both the CB15 and CB15Δ*creS* strains caused a reduction in SQ (Fig 1D and S1B Table). Thus, these results suggest that cell curvature contributes to *C*. *crescentus-*mediated plant growth enhancement, albeit other *Caulobacter* species that lack the *creS* gene (i.e., *C*. *flavus* RHGG3) have been shown to enhance plant growth [26].

### Elevated *cyoB* gene expression and media composition explain CB13-mediated seed germination inhibition for *Arabidopsis* seeds

Previously, we demonstrated that CB13 inhibited *Arabidopsis* seed germination more than any other *Caulobacter* strain we assayed (PGP or non-PGP) but still significantly enhanced plant growth relative to control conditions [22]. Given that a critical oxidative window is necessary to induce *Arabidopsis* seed germination [29], we hypothesized that CB13 may exhibit increased *cyoB* (presumptive betalain biosynthesis function) gene expression relative to other PGP *Caulobacter* strains, which would suggest that CB13 may dampen the ROS levels below the optimal oxidative window [29]. Additionally, we hypothesized that CB13 seed germination inhibition may be media-specific and concentration dependent since bacterial end-products have been shown to affect seed germination [42].

**Fig 3:**
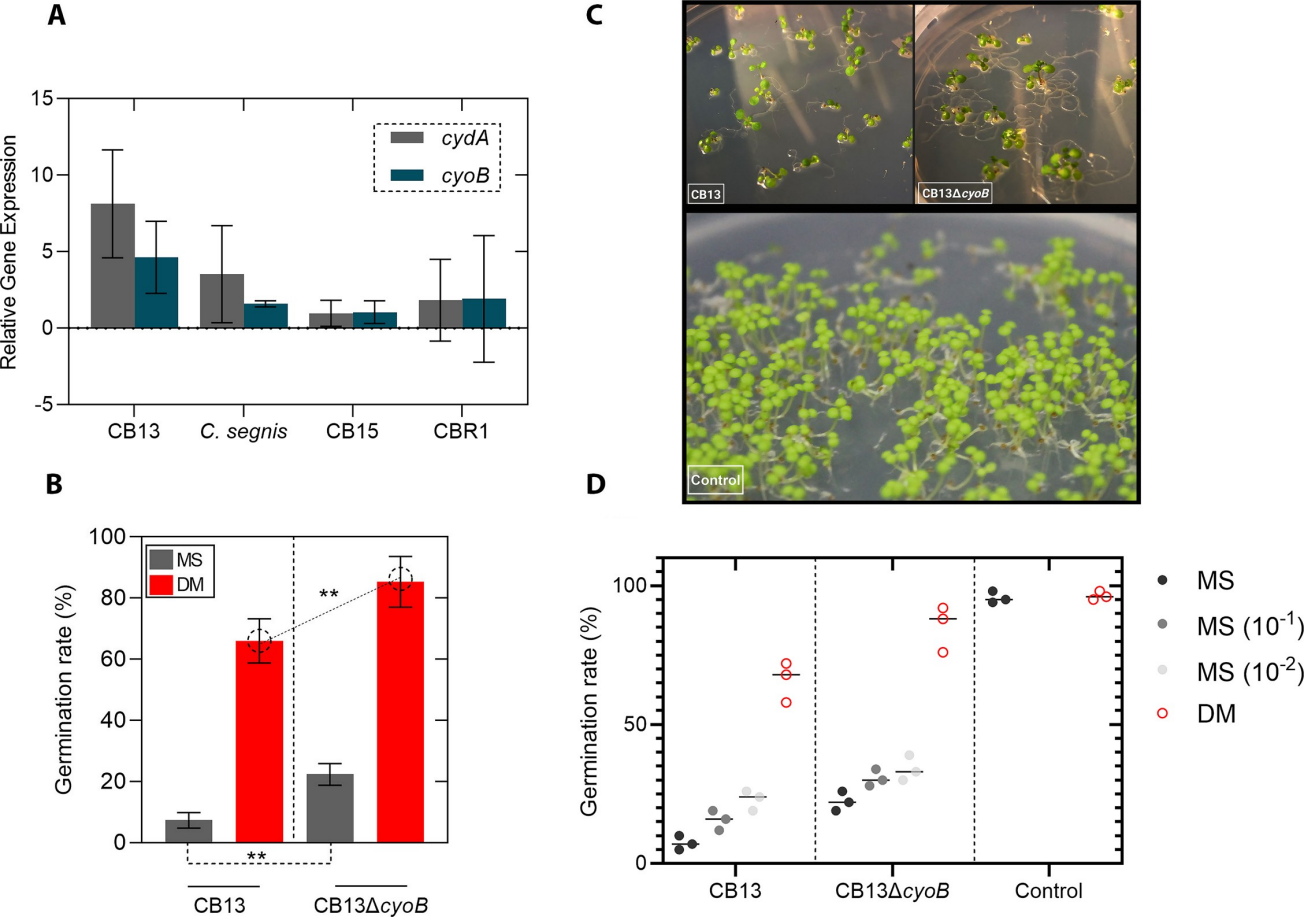
Effects of *cyoB* mutation and media composition on *Arabidopsis* seed germination. **A)** Relative gene expression of *cyoB* and *cydA* demonstrating the elevated expression of these genes by CB13 relative to other *Caulobacter* strains. Expression levels were determined using *rho* as the internal standard, and Δct values are displayed. Bars denote variance between independent replicates. **B)** Germination rate comparisons between experimental conditions are represented. Bars denote replication variances, and p-values were derived using a Welch’s t-test (** = p ≤ 0.001). A total of 50 seeds per condition were used in each independent replicate (n = 3). **C)** Seedlings grown in the presence of either sterile tap water (control), CB13 cells, or CB13Δ*cyoB* cells on defined media (DM; see Materials and Methods). Photos were captured at 18 days after seed plating (14 days after transfer to the environmental chamber). **D)** Germination rate comparisons (media composition and *cyoB* gene knockout effects) for CB13 and CB13Δ*cyoB* experimental conditions are displayed (MS = Murashige and Skoog; DM = defined media). Each dot corresponds to an independent experiment (S4 Table).

To test our first hypothesis, we performed RT-qPCR to determine the relative expression of two genes with predicted functions involved in betalain synthesis (*cyoB* and *cydA;* EC 1.10.3-) and found that the *cyoB* and *cydA* genes of CB13 were expressed at significantly higher levels than those of *C*. *segnis* (PGP *Caulobacter* strain that moderately decreases *Arabidopsis* germination rates but enhances plant growth) (Fig 3A). To address any species-specific differences regarding gene expression, we also quantified the relative gene expression of these genes in two additional PGP *Caulobacter* strains that enhanced seed germination rates (*C*. *crescentus* CB15 and *C*. *crescentus* CBR1), and we observed that CB13 also expressed the *cyoB* and *cydA* genes at higher levels than those observed in these strains (Fig 3A and S3 Table). Next, bacterial cultures were used to inoculate sterile *Arabidopsis* seeds and germination rates were measured 7 days post inoculation (DPI). We reasoned that since CB13 exhibits relatively high ROS scavenging related gene expression compared to other PGP strains, the *Arabidopsis* seeds that were inoculated with the knockout mutant cells (CB13Δ*cyoB*) would have increased germination rates relative to seeds inoculated with CB13 cells. Consistent with our hypothesis, we observed that seeds inoculated with CB13Δ*cyoB* cells germinated at a rate of ~5-fold greater than did those inoculated with CB13 cells (7 DPI), and lateral root formation was increased relative to those in the CB13 inoculum condition at 18 DPI (Fig 3B and 3C). In contrast, differences in germination rates between the *C*. *segnis* and *C*. *segnis*Δ*cyoB* inoculum conditions were not observed (S4 Table), which is consistent with the elevated expression of *cyoB* that we observed in CB13 cells. Given that CB13 and CB13Δ*cyoB* cells appeared to grow similarly on MS plates with *Arabidopsis* seeds (S1 Fig), the increased *cyoB* gene expression that we observed in CB13 (relative to other conditions) may play a role in dampening the oxidative window below the optimal concentrations that drive *Arabidopsis* seed-to-seedling transitions.

To test whether the growth medium impacted CB13-mediated seed germination, we first plated CB13 inoculated *Arabidopsis* seeds on standard MS plates (pH adjusted to 7.5) and defined media (DM) plates (0.5 mM MgSO_4_ + 1 mM CaCl_2_ + 1.5% Bacto agar) and calculated relative germination rates at 7 DPI. Our results suggested that the ability of CB13 cells to inhibit seed germination is media-specific since germination rates were increased when seeds were plated on DM compared to MS plates (Fig 3C and 3D, S4 Table, and S2 Fig). Importantly, the media composition (MS vs. DM) did not affect the germination rates of the uninoculated seeds (Figs 3D, S1 and S2). In addition, we reasoned that CB13-mediated seed germination inhibition (on MS plates) would be contingent on bacterial cell concentration. To address this idea, we inoculated *Arabidopsis* seeds with discrete concentrations of CB13 cells (OD600_nm_ = 1.0, 0.1, 0.01) and observed that a decrease in CB13 cell concentration led to an increase in *Arabidopsis* seed germination rates on MS plates. In contrast, differing CB13 cell concentrations did not appear to alter *Arabidopsis* seed germination rates when they were grown on DM plates (S4 Table). Moreover, seeds that were inoculated with CB13Δ*cyoB* cells showed increased germination rates and enhanced root growth on each media type (MS and DM) compared to seeds that were inoculated with CB13 cells (Fig 3C and 3D and S4 Table). To determine the degree to which these two variables (media composition and *cyoB* function) contribute to the CB13-mediated seed germination inhibition, we analyzed this dataset using a two-way ANOVA. Our results suggested that media-composition addressed ~80.0% of the germination inhibition, while 15.0% of the variation was explained by the impact of the knockout mutation and the remaining ~2.0% (~3.0% uncertainty) was explained by interactions between the two variables (S5 Table). Thus, both elevated *cyoB* gene expression and the seed plating media composition contribute to the CB13-mediated seed germination inhibition that we previously observed [22].

### CB13 may inhibit *Arabidopsis* seed germination by lowering local pH concentrations

**Fig 4:**
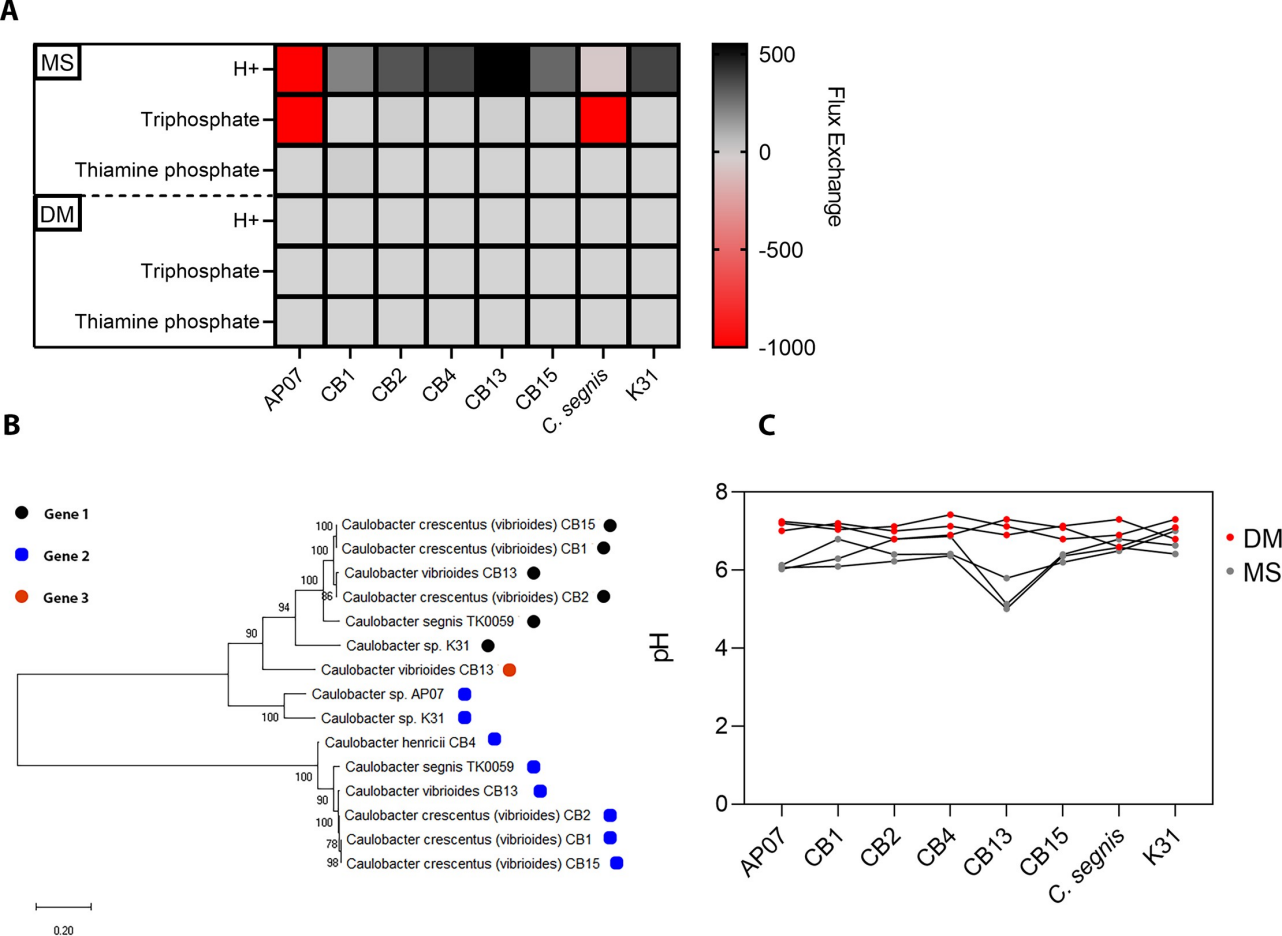
Genomic mining for metabolite associations. **A)** Heatmap of ModelSEED Flux balance analysis values depicting the unbuffered abundance of H^+^ ions theoretically generated by CB13. **B)** Phylogenetic tree comparing the predicted amino acid homology of the multiple 2,5-dioxovalerate dehydrogenases (aldehyde dehydrogenase; EC 1.2.1.26) found in *Caulobacter* genomes. Amino acid sequences were aligned using CLUSTAL in MEGAX (Jones-Taylor-Thornton Model), and bootstrap values (1000X) are shown on branches. Branch lengths correspond to amino acid substitutions per site. **C)** Line plot of measured pH values derived from post-incubation cultures (11 DPI) of select *Caulobacter* strains grown in defined media (DM) and Murashige and Skoog (MS) media.

Since our seed plating assay results indicated that CB13-mediated germination inhibition is significantly linked to the media-specific component, we leveraged the PATRIC 3.6.7 database to construct a flux balance analysis (FBA) metabolome model (ModelSEED) that predicts the relative H^+^ ions exchanged (byproducts of nutrient cycling) in the environment (MS media) for each of the experimentally tested *Caulobacter* strains (Fig 4A). Our results suggested that CB13 harnesses the potential to yield more H^+^ ions than any of the other *Caulobacter* strains that we analyzed (AP07, CB1, CB2, CB4, CB15, *C*. *segnis* TK0059, K31), and the increase of H^+^ ion flux would likely not be buffered since phosphate fluxes were predicted to remain relatively constant (Fig 4A and S6 Table). In contrast, when we reconstructed the metabolomic potential for CB13 using DM + glucose as the substrate, the H^+^ ion flux substantially decreased (S6 Table). Given that our FBA factored in substrate availability (MS and DM media) and reaction stoichiometry, it is likely that the results gained from our metabolic reconstruction analyses reflect those of our seed plating assays (Fig 3D). In addition, compared to the genomes of other *Caulobacter* strains, the CB13 genome harbors an additional predicted gene product that codes for an aldehyde dehydrogenase enzyme (2,5-dioxovalerate dehydrogenase; EC 1.2.1.26) (Fig 4B), which renders H+ ions as a result of its catalytic activity (carbohydrate metabolism). To test the results derived from our computational analyses, we measured the pH of bacterial cultures grown in MS media and DM media (+1% glucose to adjust for the carbon source that germinating seeds provide) at 11 DPI. Consistent with our FBA analyses, CB13 decreased the pH in the MS media below that of the other *Caulobacter* strains. In contrast, significant pH reductions in the DM media were not observed for any strain (Fig 4C and S7A Table). Further, when we tested the pH directly surrounding the developing seedlings on MS media (11 DPI), we observed that seedlings inoculated with CB13 and CB13Δ*cyoB* cells were surrounded by a pH of ~6, whereas all other conditions maintained a pH of ~7–8 (S7B Table). Moreover, when we artificially increased the local pH concentrations surrounding the developing seedlings (pH 7.5 to pH 10), we observed that both CB13 and CB13/CB13Δ*cyoB* inoculated seed conditions decreased the local pH concentrations (from pH 10 to 9), whereas the other conditions maintained a pH of 10 (S7B Table). As a result, CB13 and CB13Δ*cyoB* strains enhanced germination rates relative to neutral pH conditions and control conditions (S3 Fig). Since low pH has been linked to reduced *Arabidopsis* seed germination rates [43–45], it is plausible that the additional 2,5-dioxovalerate dehydrogenase encoding gene in the CB13 genome may (in part) contribute to CB13-mediated seed germination that we observed under neutral pH conditions.

## Discussion

The advent of large-scale omics projects has catapulted our understanding of which bacterial genera tend to associate with plants, and recent studies have begun to hone our knowledgebase regarding the functional prerequisites of these plant-bacteria interactions [22,46]. However, many outstanding questions remain concerning the functional factors that many plant-growth-promoting bacteria (PGPB) provide to their host(s). Here, we elucidate two underlying genetic factors (*cyoB* and *creS*) that contribute to *Caulobacter*-mediated plant growth enhancement (increased biomass), and provide computationally-derived factors that may explain the seed germination inhibition that we previously observed in our plant growth system [22].

Although the key molecular mechanisms that drive the interactions between PGP *Caulobacter* strains and *Arabidopsis* remain outstanding, our study demonstrates that a functional *cyo* operon is required for select PGP *Caulobacter* strains to enhance the growth of *Arabidopsis* plants. Moreover, given the predicted function(s) of the *cyo* operon our data suggest that ROS scavenging activities might impact positive interactions between PGP *Caulobacter* strains and *Arabidopsis*. However, the detailed mechanisms that govern the crosstalk between select PGP *Caulobacter* strains and *Arabidopsis* in the context of ROS scavenging abilities remain unknown. Therefore, future investigations will be aimed at understanding if and to what degree select PGP *Caulobacter* strains can regulate ROS levels in *Arabidopsis* plants to ultimately enhance plant growth. Nevertheless, it is well-established that in plants (as in other organisms) ROS develop as a result of aerobic metabolism, and they can cause irreversible DNA damage leading to cell death or alternatively drive important signal cascades that subsequently regulate normal plant growth and development [47,48]. Thus, ROS molecules must be kept in balance to maintain plant biochemical and physiological states. Given that plants and microbes have coevolved for millions of years [1], orchestrated processes (between plant and microbe) that maintain the balance of ROS have likely undergone functional selection.

**Fig 5:**
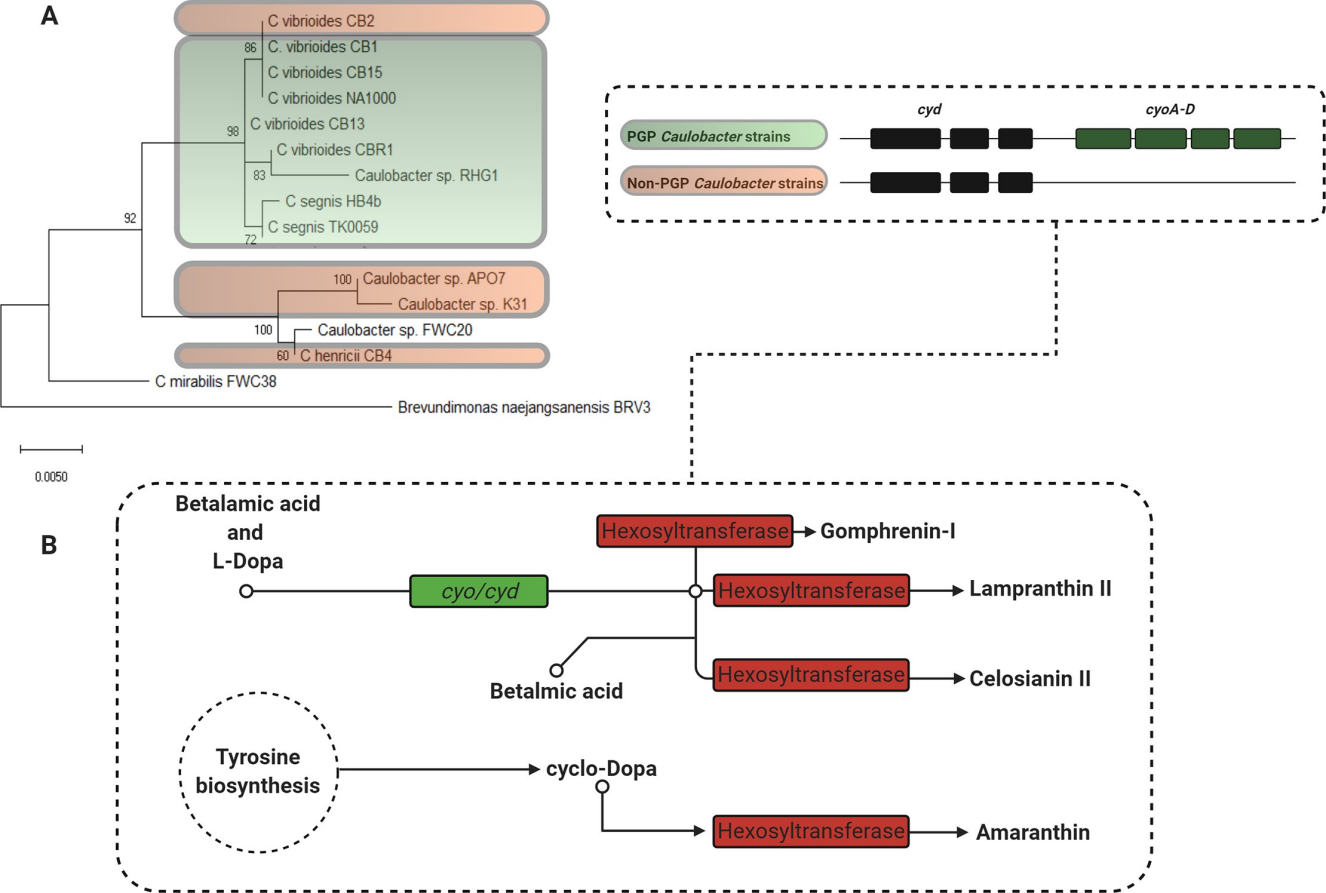
Simplified cartoon of betalain biosynthesis among *Caulobacter* strains. **A)** Phylogeny of various *Caulobacter* strains based on 16S rDNA sequences. Strains harboring both the *cyo* and *cyd* operons are highlighted in green (PGP strains), whereas strains with only the *cyd* operon are highlighted in red (Non-PGP strains). Nucleotide sequences were aligned using CLUSTAL in MEGAX (Tamura-Nei Model), and bootstrap values (1000X) are shown on branches. Branch lengths correspond to nucleotide substitutions per site. **B)** Gomphrenin-I, lampranthin II, and celosianin II function as betalains, while amaranthin functions as a lectin with betacyanin properties. *cyo* (EC 1.10.3-) corresponds to the operon (*cyoA-D*) that is unique to PGP *Caulobacter* strain genomes, and *cyd* (EC 1.10.3-) corresponds to the *cyd* operon that is conserved among the *Caulobacter* strains we previously analyzed [22]. Hexosyltransferase facilitates the conversion of several betalains and the lectin, amaranthin.

In a previous paper, we proposed that ROS scavenging might be a PGP factor that select *Caulobacter* strains employ to enhance plant growth since they contain an extra cytochrome ubiquinol oxidase operon and the proteins produced from both the *cyo* and *cyd* operons can contribute to ROS scavenging [22]. Previous studies linked gomphrenin-I—a type of betalain—to high ROS scavenging activity [28] and suggested that even under optimal plant growth conditions additional ROS scavenging activity supplied by the local microbiome could modulate plant growth through development stages [47,49,50]. Given that PGP *Caulobacter* strains harbor the genomic architecture (i.e., *cyo* and *cyd* operons) to potentially biosynthesize multiple betalain types (Fig 5) and do not depend on the functionality of the *cyo* operon for survival (Fig 2), the *cyo* operon may indeed confer PGP *Caulobacter* strains with fitness benefits that could be deemed advantageous in plant-microbe contexts. Consistent with these predictions, when we disrupted the cytochrome ubiquinol oxidase *cyoB* gene, the resultant strain had lost its ability to enhance the growth of *Arabidopsis* (Fig 1). We also predicted that disruption of the *cyo* operon would not impair the function of the electron transport chain since some *Caulobacter* strains contain only the *cyd* operon [22]. Our bacterial cell growth assays (Fig 2A) and re-isolation data (Fig 2B and S2 Table) support this hypothesis since no differences were observed when the growth rates of the *cyo* knockouts were compared to those of their parent strains. However, we acknowledge that differences (e.g., plant root colonization ability) between parental and mutant strains could have persisted *in vivo* as a function of plant development, which our bacterial cell growth assays and re-isolation experiments would not have captured.

The *cyo* operon predicted protein sequences (*cyoA-D*) in the genomes of both CB13 and *C*. *segnis* TK0059 share significant amino acid homology (>60%) with those of various bacterial genera, and a few of the strains within these genera have been isolated from plant microbiomes (S8 Table). The *cyo* operon also includes three additional genes, one annotated as a SURF1 family gene that would assist in cytochrome oxidase complex assembly and two genes that code for a sensor histidine kinase and its corresponding receptor. This gene arrangement is a conserved feature of the *cyo* operons found in PGP *Caulobacter* strains whose genomes represent all three branches of the *Caulobacter* phylogenetic tree (Fig 5). Since the sensor histidine kinase and receptor genes are distal to the *cyoB* gene, the disruption of the operon in our constructs may have eliminated the expression of these downstream genes. Therefore, the loss of sensor histidine kinase expression in the *cyoB* mutants could contribute to the inability to enhance plant growth. Moreover, we did not investigate the functional consequence(s) of direct mutations to the *cyoA*,*C*,*D* gene(s), nor did we employ mutant phenotype rescue experiments (i.e., complementation); therefore, further investigations should be targeted toward understanding the functional role(s) of each gene in the *cyo* operon in the context *Caulobacter*-mediated plant growth enhancement. Nevertheless, these experiments indicate that a functional *cyoB* gene is required for both *C*. *vibrioides* CB13 and *C*. *segnis* TK0059 to enhance the growth of *Arabidopsis*.

Our previous observations [22] suggested that the interactions between developing *Arabidopsis* seedlings and CB13 cells were complex since CB13 cells significantly decreased seed germination but subsequently enhanced plant biomass (data collected roughly six weeks post germination). And, given the high degree of genomic synteny among the PGP *Caulobacter* strains we analyzed [22], we reasoned that variations in redox related gene expression among the strains may provide insight regarding these complex interactions since ROS are critical during seedling development [29,35–39]. To test our hypothesis that expression of the *cyo* operon might explain the severe decrease in seed germination that we observed for CB13 inoculated seeds, we plated *Arabidopsis* seeds with either CB13 cells or CB13Δ*cyoB* cells and calculated germination rates. Consistent with our hypothesis, the *cyoB* loss-of-function mutation facilitated an increased germination rate for *Arabidopsis* seeds (Figs 3B and S1 and S2), and the resultant seedlings developed slightly longer roots and more root hairs relative to those inoculated with CB13 cells (Fig 3C), which is in agreement with previous reports that showed that increased ROS concentrations can increase root length and root hair formation [32,51].

Although a functional *cyoB* gene partially explained the CB13-mediated seed germination inhibition that we observed, germination rates still appeared diminished compared to those in control conditions and other PGP *Caulobacter* strain conditions (S4 Table). To establish a theoretical framework for CB13-mediated inhibition of seed germination, we performed a metabolomic reconstruction analysis of the CB13 genome and determined that growth of CB13 might lower the pH of the surrounding microenvironment. When we measured the pH of cultures and the proximal zones surrounding developing seedlings (11 DPI), we found that, as predicted, CB13 produced more acid than any other strain, which lowered the pH in the surrounding environment (Fig 4C and S7A Table). After artificially increasing the pH surrounding the developing seedlings (from 7.5 to 10), we also observed that both the CB13 and CB13Δ*cyoB* inoculated seeds germinated at faster rates than they did under neutral pH conditions (S3 Fig). The pH concentrations surrounding the seedlings also dropped to ~9 in both the CB13 and CB13Δ*cyoB* inoculated conditions, whereas each of the other conditions remained at a pH 10 (S7B Table), which suggests that the expression of the *cyoB* gene does not impact acid production, and CB13 inhibits *Arabidopsis* seed germination (in part) by lowering the surrounding pH. Next, we plated *Arabidopsis* seeds on defined media (DM) plates where only limited growth could occur (S1 and S2 Figs) and observed that seed germination in the presence of CB13 was greatly improved (Fig 3D). However, abundant bacterial growth alone likely does not explain germination rate inhibition by CB13 since seeds inoculated with varying concentrations of CB13 cells did not appear to impact seed germination on DM plates (S4 Table), and seeds inoculated with *C*. *segnis* cells germinated efficiently despite developing in the presence of abundant bacterial growth (S1 Fig). Taken together, our observations are consistent with several reports that link low pH to decreased germination rates [43–45]. However, other reports [52,53] have linked low external pH to faster germination rates, and external pH changes have also been shown to modulate IAA production, pectinase activity, and iron uptake gene expression [52–55]. Therefore, the interplay between pH and several signaling pathways probably impacts seed germination in variable and complex ways.

Another functional insight that we gleaned from our experiments was the impact that cell curvature had on PGP ability (Fig 1). Using a mutant strain unable to form curved or ‘crescent’ shaped cells [40], we demonstrated that the loss of cell curvature reduced the ability of *C*. *crescentus* CB15 to enhance plant growth (Fig 1 and S1A and S1B Table). It is highly unlikely that cell curvature alone is the causal factor for *Caulobacter*-mediated plant growth enhancement since some PGP *Caulobacter* strains lack the *creS* gene [26]. A functional *creS* gene may, however, facilitate the presumed proximity-dependent requirement for PGP factors (i.e., a functional *cyoB* gene) if bacterial cell attachment to root structures is a prerequisite for *Caulobacter*-mediated plant growth enhancement [23], but these microscale interactions (e.g., endosphere vs. rhizosphere colonization dynamics) remain to be tested. Nevertheless, recent evidence suggests that cell curvature may provide a selective advantage for niche adaptation in select contexts [40]. Additional findings have also demonstrated that cell shape, cell wall composition, and motility factors may function as valuable proxies for estimating species abundance across environmental gradients [34], albeit the exact mechanistic factors governing these host-microbe interactions have been relatively unexplored. Nonetheless, the cell curvature of CB15 cells appears to facilitate their ability to enhance plant growth, but cell shape is not a sole determinant of *Caulobacter*-mediated plant growth enhancement since our previous analyses demonstrated that plant growth enhancement is not a conserved feature among *C*. *crescentus* strains [22].

Taken together, these results suggest that PGP bacteria have a complex relationship with their plant hosts and the elucidation of these relationships requires careful experimentation under controlled conditions.

## Materials and methods

### Bacterial growth conditions

Overnight cultures were grown in peptone yeast extract (PYE) [56] and were derived from frozen stocks. Each culture was viewed with a phase-contrast microscope to check for contamination prior to experimentation. For low aeration growth curve assays, cells were cultured overnight, and cell cultures (mid-log phase) were then diluted 100-fold to a final volume of 10 mL with a surface area to volume ratio of 0.1:1.0. Subsequent cultures were placed in an orbital incubator shaker set to 100–150 rpm. Optical densities were collected using a Klett-Summerson photoelectric colorimeter. Growth curve assays were performed three times independently, and values are reported as Klett and cells per milliliter. In addition, overnight cultures were also streaked on PYE plates, and subsequent colony growth was observed at 24- and 48-hours post-incubation. To determine pH concentrations of the assayed cultures, bacterial cultures were grown in Murashige and Skoog (MS) [57] and defined media (DM) (1 mM MgSO_4_ + 0.5 mM CaCl_2_) supplemented with 1% glucose for 11 days and pH values were determined using a pH probe (S7A Table).

### Plasmid construction and bacterial mutant generation

The plasmid used to generate *cyoB* mutants was commercially constructed (GeneScript), and it was used to generate gene knockouts via homologous recombination. Briefly, ~250 bp of the *cyoB* flanking regions were cloned into the vector pUC57-Kan at *Pfo*I and *Nde*l (left flanking region) and *Bsa*Xl and *PfI*lll (right flanking region) sites. Electrocompetent cells were prepared by resuspending mid-log phase cultures [56] in 30% glycerol, and the pUC57-Kan-cyoB vector was electroporated into either *C*. *vibrioides* CB13 or *C*. *segnis* TK0059 cells using a Bio-Rad Gene Pulser (2.5 kV, 25 μF, 400 Ω). Subsequently, 1 mL of PYE was added to each electroporated strain, and the resultant cell suspensions were grown for three hours at 30°C with aeration. Afterwards, cell cultures were plated on PYE+ kanamycin (50 mg/L) agar plates and incubated at 30°C for 48–72 hours. Single colonies were aseptically streaked onto PYE + kanamycin plates, and a single colony from each plate was grown in PYE broth to generate pure cultures for DNA extraction (DNeasy Blood and Tissue Kit). To confirm that the anticipated homologous recombination events occurred without a tandem insertion of exogenous DNA (i.e., the mutant strain constructs did not harbor the wildtype allele) in the *Caulobacter* strain genomes, mutant strain DNA was subjected to PCR using the following primer pairs: *cyoBFWD* (5’- TTTGAATTCCCTGTTCTTCGCCTGGAAGT-3’), *cyoBREV* (5’-TTTTTTCTCGAGACCAGAGCGATGAAGCTCAA-3’), 16sFWD (5’-GGTTACCTTGTTACGACTT-3′), and 16sREV (5’-GTGCCAGCMGCCGCGGTAA-3′), and subsequent Sanger sequencing was employed to validate the sequences (both the 16s rDNA and the *cyoB*-Kan insert). The cell curvature mutant (CB15Δ*creS*) was obtained from Zemer Gitai’s laboratory at Princeton University.

### Plant growth experiments

All plant growth assays were conducted as previously described by Berrios and Ely (2020) [22]. Briefly, bacterial cultures were grown overnight and were then pelleted and rinsed (3X) with sterile tap water to remove residual metabolites. Culture concentrations were adjusted to an OD600_nm_ = 1.0, and sterilized *Arabidopsis* seeds (Ler-O) were inoculated with 500 μL of the bacterial culture (depending on the condition). Control seeds were inoculated with 500 μL of sterile tap water. Seed mixtures were incubated at room temperature for 30–45 minutes and were plated on either Murashige and Skoog (MS) or defined media (DM: 1 mM MgSO_4_ + 0.5 mM CaCl_2_ + 1.5% Bacto agar) with pH conditions adjusted to 7.5. The plated seeds were stratified for 4 days at 4°C and were transferred to an environmental chamber (16:8 light/dark photoperiod) under a light intensity of ~150 μM/m2/s. Germination rates were calculated (total number of germinated seeds divided by the total number of plated seeds; n = 50) at 7 DPI, and the pH concentrations surrounding developing seedlings were derived using ADVANTEC^®^ Whole Range pH test strips (TOYO ROSHI KAISHA, LTD.) at 11 DPI. Seedlings along with any ungerminated seeds were transferred aseptically from MS plates to sterilized soil in pre-washed plastic trays (3 X 4 grid), and plastic domes were placed over each tray to increase humidity for the first week and then the domes were removed thereafter. The plants were bottom watered as needed (1–2 times per week) with sterile tap water for 5–6 weeks. Each experiment was conducted twice (24 plants per condition), which yielded a final dataset of 48 plants per condition. Fresh plant weight (PW), inflorescence height (IH), basal rosette diameter (BRD), silique quantity (SQ) data, root architecture, and bacterial cell re-isolation data were collected for each sample as previously described [22]. One-way ANOVAs and Welch’s t-tests were performed to determine significant differences within and between conditions.

### RNA extraction and RT-qPCR

Bacterial cultures were grown in PYE to mid-log phase (rotational incubator at 30°C). RNA was extracted using a Qiagen RNeasy kit according to the manufacturer’s protocols. The forward and reverse primers that were used to measure *cyoB* gene expression in both wildtype and mutant constructs were 5’-CAACTGGCTGTTCACGATGTA-3’ and 5’-GATCACGAAGGTGACCATGAA-3’, respectively, and the forward and reverse primers that were used to measure *cydA* gene expression were 5’-TGGTCATCATGGAGAGCATCTA-3’ and 5’-ACGAAGTTGATGCCGAACAG-3’, respectively. The *rho* gene was used as an internal control, and the corresponding forward and reverse primers used for amplification were 5’-GCACGGTGAAGGGCGAGG-3’ and 5’-GAGTCC AGCAGGATGACGA-3’, respectively. Each assay was performed twice in triplicate, and relative expression (Δct) values (internal control (*rho*) compared to the target gene) are reported.

### Comparative genomics

Metabolomic reconstruction analyses of the genomes of *Caulobacter* strains were conducted in PATRIC 3.6.7 and analyzed in ModelSEED [58]. Homology-based calculations were derived from BLASTn or BLASTp for nucleotide and amino acid sequence comparisons, respectively [59]. Quantitative gene binning was performed in PATRIC 3.6.7 using subsystem and pathway functions. Gene and protein sequences were deemed homologous using E-value cutoffs of 10^−5^, query coverages of >60%, and identities of >70%.

Phylogenetic analyses were performed using CLUSTAL in MEGAX (Jones-Taylor-Thornton Model or Tamura-Nei Model). Each alignment was bootstrapped (1000X), and branch lengths depict the degree of amino acid or nucleotide substitutions among sequences. A complete list of each of the strains used in these analyses and their corresponding accession numbers can be found in S6 Table.

## Supporting information

S1 Fig Germination assays on Murashige and Skoog (MS) agar plates. (XLSX)

S2 Fig Germination assays on Defined Media (DM) agar plates. (XLSX)

S3 Fig Germination assays on Murashige and Skoog (MS) agar plates with pH adjusted to 10. (XLSX)

S1 Table A. Plant Growth Data. Plant weight (PW); Basal Rosette Diameter (BRD); Inflorescence Height (IH); Silique Quantity (SQ). B. P-values: t-test.
(XLSX)

S2 Table Bacterial cell re-isolation dataset. (XLSX)

S3 Table RT-qPCR dataset. (XLSX)

S4 Table Germination rate data. Rates were recored at seven DPI.
(XLSX)

S5 Table Two-way ANOVA: Media composition and cyoB mutation. (XLSX)

S6 Table Flux Balance Analysis (FBA). (XLSX)

S7 Table Caulobacter strains grown in defined media (DM) and Murashige and Skoog (MS) media. (XLSX)

S8 Table cyoA-D predicted protein sequence homologies to those of non-Caulobacter genera (top BLASTp match). (XLSX)
